# An analysis of the Mental Health of Black Canadians Fund: facilitators of success, challenges and recommendations

**DOI:** 10.24095/hpcdp.45.4.06

**Published:** 2025-04

**Authors:** Bukola Salami, Mia Tulli-Shah, Ifrah Abdillahi, Wesley Crichlow

**Affiliations:** 1 Department of Community Health Sciences, Cumming School of Medicine, University of Calgary, Calgary, Alberta, Canada; 2 Social Determinants of Health Division, Public Health Agency of Canada, Ottawa, Ontario, Canada; 3 Youth, Crime and Justice Specialization, Faculty of Social Science and Humanities, Ontario Tech University, Oshawa, Ontario, Canada

**Keywords:** mental health, Black Canadians, service provision, funding, health equity

## Abstract

**Introduction:**

In 2018, in an effort to address the mental health inequities experienced by Black Canadians, the Government of Canada announced a CAD 10 million investment to establish the Public Health Agency of Canada’s Promoting Health Equity: Mental Health of Black Canadians Fund (MHBC). The aim of this study was to examine and document the lessons learned from the MHBC, including successes and challenges.

**Methods:**

Researchers conducted document analysis of 15 participating projects from 14organizations’ annual and final reports. Researchers then conducted interviews with representatives from nine of these organizations. An embedded case study design was used in the data collection and data analysis that included content analysis of annual and final reports, as well as thematic analysis of individual interviews.

**Results:**

Analysis of the data from annual and final reports and interviews illuminated three main themes: facilitators of successes; challenges; and lessons learned and recommendations for funders. Facilitators included honorariums and incentives, participatory action research design and Black leadership. Challenges included delays (for obtaining ethics approval and program implementation); impacts of the COVID-19 pandemic; and difficulties maintaining partnerships. Finally, the lessons learned and recommendations that emerged for funders were that there is a need for longer term and more flexible funding, more Black representation and leadership within funding organizations and greater support of antiracist practices among mainstream service providers.

**Conclusion:**

The findings of this study present the challenges and opportunities in supporting work aimed at improving the mental health and well-being of Black people in Canada.

**Highlights**

- Facilitators of success among participating organizations included honorariums and incentives, participatory action research design and Black leadership.
- Challenges included delays (for obtaining ethics approval and program implementation), impacts of the COVID-19 pandemic and difficulties maintaining partnerships.
- Recommendations that emerged were that funders should provide longer term and more flexible funding, that more Black representation and leadership is needed within funding organizations and that there should be greater support of antiracist practices among mainstream service providers.

## Introduction

Black Canadians experience mental health care inequities in Canada. Poorer health outcomes may be attributed to inequities in social determinants of health, including income disparities and racial discrimination.^1^ Racialized mental health inequities in Canada have more recently gained attention from service providers and policy makers. Mental health inequities have been connected to pervasive systemic racism,^2^ corresponding inequities related to housing, income inequities^3^ and racialized barriers to mental health care and promotion services.^4^ Lack of representation in mainstream healthcare and a systemic absence of mental health care for Black Canadians has left many without supports.

In 2018, the Government of Canada announced a CAD 10 million investment to the Public Health Agency of Canada to establish the Promoting Health Equity: Mental Health of Black Canadians Fund (MHBC). The MHBC delivered a grants and contributions program to provide support to community-based projects to develop culturally focussed mental health programs in Black communities across Canada over a five-year period from 2018 to 2023, and was later extended to 2024. The organizations funded through this program promoted mental wellness through mentorship and community-based interventions. The program features three key pillars: investing in community, strengthening data and evidence, and mobilizing knowledge. Key principles include a focus on anti-Black racism, leadership by Black Canadians, evidence-based research and programming, a social determinants of health approach to program delivery, a health equity lens, cultural competence and safety, and emphasis on partnerships and collaboration.^5^

The MHBC supports community-led projects focussed on promoting the mental health of Black Canadians through two funding streams. The Incubator Stream provided short-term funding to support capacity-building activities that help community and university organizations design, develop, implement and evaluate projects that promote mental health and address its determinants for Black Canadians. The Implementation Stream provided multiyear funding to support the implementation and evaluation of community-based projects that promote mental health and address its determinants for Black Canadians.^5^

The aim of this work was to examine and document the lessons learned from the Public Health Agency of Canada’s (PHAC) Mental Health of Black Canadians Fund. Our intent is to inform future policy and program development in the areas of health equity and mental health for diverse Black communities in Canada. The study was guided by the following questions:

- 1. What have funded organizations reported as successes, facilitators and challenges of the PHAC Mental Health of Black Canadians Fund?
- 2. What are the perspectives of funded organizations, working group members and PHAC on the successes, challenges, lessons and opportunities for further refinement of the approaches used throughout the PHAC Mental Health of Black Canadians Fund?

## Methods

***Ethics approval***

Ethics approval was obtained from the University of Alberta Research Ethics Board 1 (Pro00130072).

***Data collection***

We sought to analyze the MHBC through two stages of data collection. First, researchers conducted document analysis of 15 participating projects from 14 participating organizations’ annual and final reports. Of the 15 projects, nine representatives agreed to participate in interviews. In the second stage, researchers conducted interviews with representatives from these nine organizations. All organizations involved in the MHBC program were invited to participate. The nine that did were representative of the larger group of funded organizations and included community and university organizations and provided geographic representation.

***Study design and analysis***

Researchers used an embedded case study design that used content analysis of annual and final reports as well as thematic analysis of individual interviews. An embedded case study design involves a researcher considering each aspect of a larger case.^6^ Therefore, researchers explored each organization in isolation before comparing all interviews and analysis of reporting documents, to understand the broader impacts of the MHBC. 

***Document review and analysis***

As a first stage in gauging organizations’ experiences with the MHBC, researchers reviewed annual reports of 15 participating projects from 14 organizations’ that received funding through the implementation stream of the MHBC. Six of these organizations (organizations 001 through 006) provided annual reports for three years (2019/20, 2020/21 and 2021/22). Two organizations (007, 008) provided annual reports for 2019/20 and 2020/21. The other seven organizations (009–015) provided one-year reporting, all for the year 2021 to 2022. Three organizations also provided final reports (001, 007, 008).

To consider each report and the entirety of the reporting provided, researchers used content analysis to map each organization’s reporting. Items on reporting grids included “Please describe some key highlights or achievements of the project during the reporting period (e.g. innovations, new partnerships, etc.)” and “Please describe any difficulties or challenges that your project encountered during this reporting period, how these were addressed and the lessons learned (e.g. reaching priority populations, governance issues, recruiting or working with partners, administrative barriers, etc.). Report contents included information about key successes, challenges, facilitators of success and recommendations for funders. 

Information on each topic was captured and tracked as its own individual code (for example: “Organization 001 - key successes - Honorariums and incentives). We then compiled all, for example, honorarium and incentives codes together, to track how many organizations focussed on each topic and what themes arose. This technique required researchers to analyze messages, meanings and themes within and across a set of documents (the 14 organizations’ annual and final reports).^7^ Looking at the entirety of this information, we found themes across facilitators of success, challenges and recommendations for future funding. 

***Interviews***

At the second stage of data collection, the project’s Principal Investigator (BS) conducted individual interviews with nine representatives from organizations that received funding through the MHBC (002, 004, 005, 007, 008, 010, 012, 013, 015). The researchers provided a letter of invitation to staff of the Public Health Agency of Canada, which was distributed to representatives of each of the funded organizations. Interested participants contacted researchers to complete interviews. Prior to interviews, all participants completed the informed consent process. Interviews took approximately one hour and were conducted over Zoom. They were audio-recorded and transcribed verbatim. Interviews used a semistructured interview guide. 

***Data analysis***

Data collection and data analysis occurred concurrently. After the data were transcribed, the researchers familiarized themselves with the data through repeated reading of text and listening to the audio recording. These data were thematically analyzed.^8^ Two members of the research team (BS and MT) developed preliminary codes, after which they met to review and agree on a coding framework. The key topics in the annual and final reports (key successes, challenges and facilitators of successes) were used as preliminary major codes, with subcodes under these codes. A member of the team then coded the data using NVivo qualitative software version 14 (Lumivero LLC., Denver, CO, US). The codes were revised, collapsed and expanded based on emerging data. Codes were refined and translated into themes as data was further analyzed and interviews were considered alongside one another.

Throughout the process, we wrote memos and notes to record our emerging awareness, including connections between themes and codes. This allowed us to further define, describe and name the themes. Data from interviews were then compared to data from annual and final reporting, and researchers identified where themes overlapped. The themes and our report were shared with all members of the team for feedback.

## Results

Analysis of the results from annual and final reports and interviews illuminated three main themes: facilitators of success; challenges; and lessons learned and recommendations for funders. Within these themes, subthemes offered insight into specific factors that shaped organizations’ experiences during their funding period. A summary of these findings is presented in Table 1.

**Table 1:** Summary of facilitators of success, challenges and recommendations for funding, Mental Health of Black Canadians Fund, 2018 to 2024

Themes	Examples	Implications for funding and policy
Facilitators of success	Honorariums and incentives	Despite the importance of honorariums in encouraging participation in programming and health interventions, a lack of support was reported. Participants identified barriers from the funder in providing honorariums to participants.
	PAR research design	PAR design allowed organizations to better respond to changing community needs during program delivery. Funder support of PAR design was a benefit of the MHBC, especially during the pandemic.
	Black leadership	Support of Black leadership and diversity in racial, gender and sexuality representation enabled organizations to tailor program design and recruitment to their client populations and embed Afrocentric principles throughout programming.
Challenges	Delays	Delays were related to pandemic interruptions and long waits to obtain ethics approvals. PHAC’s Research Ethics Board helped process one organization’s ethics submission when university delays were too long. This shows potential for funders to assist organizations experiencing delays at this stage of research.
	Impacts of the COVID-19 pandemic	Service interruptions, the move to online programming and the need to redesign programming delayed service provision to populations already feeling pandemic impacts particularly starkly. Funders may offer greater support during future crises.
	Structural violence and community-specific challenges	Systemic racism, including over-policing, lack of affordable housing, homophobia and systems of gender oppression shaped the mental health needs of organization’s client populations. Some organizations suggested funders do not understand these issues, and emphasized the need for funding flexibility.
	Maintaining partnerships	Staff turnover led to breakdown of relationships and vision shifts that impacted networking and partnerships between organizations. Thus, there is a need for long-term stability within organizations for successful collaboration.
Lessons learned and recommendations for funders	Longer term and more flexible funding	Challenges associated with creating spaces for Black people where none had previously existed demand longer term and flexible funding.
	Black representation and leadership	A lack of Black representation among funders meant that even those who approached the issues with sensitivity and competency often needed aspects explained to them because they were not Black. Perceptions of being micromanaged were largely shaped by organizations’ sizes. Smaller organizations tended to relay appreciation for PHAC support. Conversely, larger, more established organizations were more critical of being managed.
	Antiracist practices among mainstream providers	Removing barriers to Black people’s access to mainstream healthcare providers should go hand in hand with ensuring Black people do not face barriers to other aspects of mental health supports. There is a need for funders to better support Black organizations.

***Facilitators of success***

**Honorariums and incentives**

The most reported facilitator of success across both annual reporting and interview data was having the organizations provide honorariums and incentives to encourage participants to attend various activities offered by projects. Eleven organizations reported either in their annual reporting or their interviews that this was a key factor that aided their success (001, 002, 003, 004, 005, 006, 007, 008, 009, 010, 012). 

Incentives usually took the form of gift cards or financial compensation, but also included certificates for program completion and access to free services. Honorariums were especially necessary to encourage participation in virtual programming, as in-person events were able to provide food, which some organizations advised was enough of an incentive to encourage participation: 

We had to look for different ways to do that [provide meals during online programming], and really, you know, making sure that folks were eating properly, so giving them gift cards so they could buy their own food, and then also giving them meaningful, not tokenistic honorariums. A lot of the youths were, you know, folks who were struggling financially, whether they’re in school or, you know, working—working survivor jobs or, you know, in households that, you know, weren’t meeting the median income. So, folks needed to be properly compensated [005]. 

In this interview, we heard how the move to online events, coupled with economic impacts of the pandemic, meant that it was even more important to provide gift cards so people could eat. However, despite the importance of honorariums in their work, some organizations perceived a lack of support from funders related to honorariums. Two organizations (005, 007) explained there were long delays in receiving reimbursement from PHAC. 

**Participatory action research design**

Another key facilitator of success was the use of participatory action research (PAR) design. This topic was discussed by six organizations during interviews. Organizations 004, 005 and 007 all explicitly discussed the use of PAR in their programming and research. Some cited the importance of peer-led programming (002, 004), an advisory committee (004, 013), the use of ambassadors to connect to community (012) and the importance of community stakeholder involvement (010) in their design. Additionally, organizations 006, 007 and 011 all included benefits of PAR approaches in their annual reports. PAR approaches enabled them to respond to community needs and achieve better research data. 

It’s a peer-led project, and so really having them invested in the work from the beginning. I think while—as I said earlier that, you know, we actually had to do a complete shift of the model we were using, and the workshops that we were going to use in community, and so when we actually had to develop our own workshops, I think while in the beginning, you know, that was a lot of work, because we essentially had to start over, but it actually worked to our benefit, because the peers were that much more invested in the work, you know, and in terms of the great outcomes that is beneficial for community [004].

As this interviewee explains, PAR was especially beneficial when organizations were forced to pivot at the onset of the pandemic. Organizations engaged in PAR were already embedded in the communities they served so were best positioned to understand and respond to changing needs. 

The use of the PAR approach also meant an initiative focussed more on knowledge mobilization. For example, the TAIBU Community Health Centre in Toronto and PHAC co-developed the Amandla Olwazi Project, which included knowledge products and a knowledge hub to mobilize knowledge on mental health of Black Canadians. The initiative has also held conferences to support knowledge mobilization. Further information is available online.

**Black leadership**

Four organizations (002, 007, 012, 014) cited in their annual reporting having a diverse and Black-led team as a key facilitator in their success. During interviews, organizations 002, 007, 010 and 012 discussed the importance of having Black leadership, and that diversity in racial, gender and sexuality representation in programming was important to program design and recruitment of participants. Embedding Afrocentric values and principles throughout project design was also shared as an important element that itself comes from having Black people in positions of leadership and decision-making within organizations (013).

I think from an organizational perspective, the work that we’ve been doing so far around framing programs with and embedding them within an Afrocentric values and principles has also been very, very helpful, because it speaks to all members of the funded project people. It brings us into a space where, you know, that unifying kind of a, you know, brainstorming and discussions, and everybody can identify with those principles, so I think that has also been very helpful [013]. 

This participant explained during the interview that the program has grounded itself in questions around the meaning of knowledge in the African context, as well as how knowledge is gathered, who participates and how it is disseminated. In another case, organization 007 explained how programming drew on poetry practices from the Democratic Republic of the Congo. These practices enabled healing through writing about family, lineage and place.

***Challenges***

**Delays**

The most reported challenge across reporting was delays with program implementation. Delays were reported as a key challenge by 11 organizations in their reporting (001, 002, 003, 004, 005, 007, 008, 010, 011, 012 and 014). Four of these organizations expanded on this topic during their interviews (002, 003, 013, 014). These delays were mainly related to obtaining research ethics approval in order to collect data from project participants. Organization 005 eventually applied through PHAC’s Research Ethics Board process when they were unable to obtain approval from a university. 

In several cases, organizations were forced to delay program launch or data collection because they could not obtain ethics approval in time for planned start dates. For example, organization 001 had to push all Year 1 activities into Year 2. Organizations 003 and 012 were able to begin programming and service provision, but data collection had to wait until the following year. 

By the time we actually get the approval [for a funding carryover request], like two months or three months has passed, right? And so, let’s say it was a five-month window, if that two months has passed by the time we actually get an approval, I’m not going to act on those projects, because what if it’s not approved, right? And so, then it kind of limits the amount of time I have to actually get the activities done, so it kind of puts a stress or strain in that regard [004]. 

This interview showed that, in this case, and in others (002, 003, 013, 014), delays in approvals to requested changes to the initial terms of the funding agreements (e.g. time period, funding amount), as well as delays in finalizing evaluation plans and funding agreements with PHAC, pushed back program activities. 

**Impacts of the COVID-19 pandemic**

A total of 10 organizations reported various COVID-19 pandemic impacts on their projects in their annual reports (001, 002, 003, 004, 005, 006, 007, 008, 009, 011). With the exception of organizations 009 and 011, organizations that reported key challenges related to the pandemic were all operating in 2019/20—the year of the onset of the pandemic and initial public health guidelines. Challenges included the need to shift programming from in-person to online, and issues related to ethics and funding. Several organizations were forced to apply for ethics amendments to move activities online, which delayed program launch dates. Some also had to submit budget change requests to PHAC to either move Year 1 funding to Year 2 or to spend money initially earmarked for space, travel, food and so on, to honorariums for online participants. 

Additionally, during interviews, eight organizations (002, 004, 005, 007, 008, 010, 012, 013) explained that impacts of the COVID-19 pandemic were a key challenge. These impacts included service interruptions, the move to online programming, redesign of programming in some cases and the fact that most organizations were serving populations that felt the pandemic particularly harshly. Pre-existing income inequities including lack of affordable housing and food insecurity became even more significant to service provision and program planning. During their interview, one organization said:

People lost their ability to even pay their rent. You know, because they didn’t have an income. Food insecurity. You know, so one of the things we had to do was we were able to, you know, we gave—you know, in our budget for the program, we had put refreshments, because that’s always an incentive as well, to come. But instead of refreshments, we gave people food vouchers, and food cards to buy groceries and buy food, and that was really important [007]. 

Additionally, organizations that served Black populations facing intersecting oppressions, for example queer Black youth, had to contend with impacts unique to their communities. For example, one organization (012) saw clients who already faced pressure to mask their faces suffer increased mental health effects of social isolation during lockdowns. 

**Structural violence and community-specific challenges**

Five organizations (004, 005, 007, 010, 013) explained broader systemic violence and inequities were a challenge in their work during interviews. Examples of such inequities are the over-representation of Black people in the criminal justice system and parallel under-representation of Black people in restorative justice programs, a lack of affordable housing, low income and systemic racism, homophobia and systems of gender oppression. In interviews, several organizations (004, 007, 010, 013) discussed how the murder of George Floyd increased mental health needs among their client populations. 

Then the George Floyd murder happened that was, you know, sort of visible to the whole world … and I think what I noticed was, there was a bit of even more anger among this group, simply because they said, “We’ve been saying this for so long, and no one listened to us. We’ve been talking about the fact that we are brutalized every—you know, by police. We’re afraid to go out of our house, because, you know, we’ll just get stopped because we’re Black, walking while Black.” Because, you know, this is a community where people are just walking on the street or waiting for a bus. They’re not necessarily driving. But it—and they’re younger folks. So, it made them angry, and they discussed it. They had the opportunity to discuss it in the group [007]. 

Acute anger and fear about police brutality following George Floyd’s murder was an important example of the demand to address community needs as they arise. This representative went on to suggest that funders may not understand issues organizations and their client populations are dealing with and therefore may be unable or unwilling to offer necessary flexibility.

**Maintaining partnerships**

Maintaining partnerships during the pandemic was cited as a key challenge by four organizations. Organizations 001, 004, 007, 010 mentioned losing partnerships in their reporting. Such breakdown was often due to staff turnover within their partner organization, leading to the interruption of relationships and project momentum. Organizations 001 and 010 reported how staff changes at partner organizations led to vision shifts and meant organizations started to pursue divergent goals. 

For example, organization 001 reported the loss of partnership and participant attendance due to their partnership with an organization that provided gender and sexuality inclusion training. In this case, inclusive programming and their partnership with a gender and sexuality inclusive organization led to the dissolution of other partnerships or prevented potential partnerships from forming. While those LGBTQ organizations that participated in interviews did not report similar problems, one representative did explain how queerness and transness is often invisibilized or deprioritized in work supporting Black client populations. Thus, they contended that collaborations to support Black LGBTQ clients can take a long time to form. 

***Lessons learned and recommendations for funders***

**Longer term and more flexible funding**

A key recommendation for funders was to provide longer term and more flexible funding (002, 004, 012, 015), and to provide infrastructure and core funding (008). As one participant explained during the interview:

We’re trying to create spaces that like almost maybe for like at least 350 years haven’t existed here, and so to think about time in that way, it’s like yeah, maybe we should be looking at five to seven years of funding, right? Because we’re trying to work at—we’re trying to work in opposition to these 350 years where Black spaces definitely couldn’t exist. Black people couldn’t even be, right? That’s part of why our mental health suffers. It’s like so I think for me, I’m always thinking about time, how to like slow down, and to be so intentional [012]. 

The magnitude of such a challenge may demand long-term and sustained funding. Additionally, some organizations explained that, although capacity increased during the funding period, it receded back to prefunding levels once their MHBC project funding ended. Funders can also consider the larger context within which Black organizations are operating in across Canada. For instance, two representatives explained how funding scarcity creates competition between organizations engaged in similar work (005, 010). Thus, there is a need for funders to provide increased financial support to Black organizations. Doing so may help support better collaboration and partnership between these organizations. 

Participating organizations also advised that administrative work could be better organized. Financial and impact objective reporting could be made clearer and easier to complete, as bureaucracy is a barrier to program success (005, 010, 015). Additionally, an organization suggested PHAC use its reputation to facilitate partnerships with institutions that are unlikely to meet with organizations (such as school boards) without this connection (002). Brokering partnerships may offer a way to support organizations after funding relationships end. 

**Black representation and leadership**

A lack of Black representation among funders meant that even those who approached the issues with sensitivity and competency often needed aspects explained to them because they were not Black. Additionally, funder expectations around data collection sometimes didn’t match organizations’ practices (005). Also, more dedicated funding of antiracist, third-party evaluation is needed (002). These challenges created the perception among some organizations that funders do not really understand the issues facing these organizations and the communities they serve (002, 005). Additionally, project requests to PHAC for non-White, third-party evaluators were not met at times. A lack of Black evaluators combined with the fact that the main evaluator contact for the organization was White was also a challenge.

You know, we do data collection, and we do it in our way…. You know, the—this particular project is part of a very, very rigorous, as you probably know, evaluation process, and so we have an evaluator who is deeply embedded in the work to ensure that, you know, the project is in fact doing the work that it said it would do. And I think that that’s a positive because it gives credibility to this work. But, you know, there’s also wonderment about other non-Black or nonracialized organizations, and do they go through the same rigor in terms of the evaluation process that we’re going through [005]. 

Here, the interview participant discussed how, although a high degree of communication between PHAC and the organization was beneficial at times, there was also the perception that funders were micromanaging the organization because it was a Black organization. Perceptions of being micromanaged were largely shaped by organizations’ sizes. Smaller organizations tended to relay appreciation for PHAC support. Conversely, larger, more established organizations felt they were being over-managed. 

**Antiracist and antioppressive practices among mainstream service providers**

Finally, interview participants advised funders to support work aimed at decreasing anti-Black racism. Removing barriers to Black people’s access to mainstream healthcare providers should go hand in hand with ensuring Black people do not face barriers to other aspects of mental health supports, such as housing (008). Along with this, funders need to better acknowledge anti-Black racism and anti-Blackness and generate disaggregated race-based data to allow for a better understanding of the link between anti-Black structural racism and social determinants of health (010, 013, 015). PHAC and other funders could consider the larger funding context within which Black organizations are operating in Canada as well, as funding scarcity creates competition between organizations engaged in similar work. Thus, there is a need for funders to better support Black organizations. Doing so may help improve collaboration and partnership between these organizations (010).

So because there are a lot of people who are working in healthcare, so PHAC really needs to look also at ways to try to make sure that individuals who haven’t had any experience even growing up with Black Canadians, they need to understand more about the culture and cultures of individuals who are non-White Canadians, and we really need to see people who are—funding being provided to Black-led organizations, to have them to be a part of that, part of getting individuals to be a little bit more familiar with their needs [008]. 

In this interview, we learned that increasing antiracist practices across health institutions, both public health and healthcare systems, is the best way to ensure Black people do not face barriers to other aspects of mental health supports across the social determinants of health and mental health, for instance, housing, income, employment, education and so on. 

## Discussion

Despite the fact that participants reported more challenges than facilitators of success, the majority described their participation in the program as a benefit. Our findings suggest the MHBC was largely successful in supporting capacity building among organizations that received funding, despite continued room to improve the program. Facilitators of organizations’ successes included the ability to provide honorariums and incentives to participants, the incorporation of PAR design during project conceptualization and data collection and the centralization of Black leadership in program delivery. 

These results support many prior findings that have asserted the benefits of incentives to encourage community participation in health interventions.^9^,^10^ However, the significance of honorariums has also generated ethical debates. Some have cautioned against the use of monetary incentives to encourage participation (see, for example Head’s concern that incentives motivated participants to agree to interviews even if they contributed nothing “useful” to the research^11^). Others have called on researchers to establish upfront rules surrounding incentives in community-based research.^12^ Neither perspective here seems to engage participants as equal and valuable co-creators of research with diverse needs. Instead, our study shows the need for reciprocal relationships between providers, researchers and community members, as well as the need to value efforts of participants.

Bottom-up approaches, such as PAR, to data collection, knowledge mobilization and service provision need not demand rigid rules around incentives and honorariums, nor be concerned with whether or not the value researchers can extract from participants is worth the cost. Instead, participants in our study explained they need the ability to pivot and adapt incentives to meet the needs of the client populations and communities they serve. Our conclusions surrounding incentives should be considered alongside our findings advocating the benefits of PAR design in supporting community-based research and service provision and funding targeting such work. A recent article noted the challenges of PAR, including risks of power inequities between researchers and participants.^13^ Failure to offer relevant, materially significant and flexible incentives may undermine PAR efforts. 

A third facilitator of success, Black leadership, enabled organizations to ground their programming in antiracist and culturally relevant principles of service provision, data collection and knowledge mobilization. This leadership enabled the incorporation of Afrocentric practices such as lineage poetry in programming. The need for representation and diverse leadership across healthcare has recently gained increased traction.^14^,^15^ We add to these calls for greater support of Black leadership in community-based mental health programming and research alongside the need for increased representation within funder organizations. 

Key challenges included delays (both related to and separate from the onset of the COVID-19 pandemic) as well as impacts of pandemic lockdowns, structural violence and community-specific challenges, and difficulties maintaining partnerships. Delays were most often tied to obtaining ethics approval but also sometimes occurred when organizations applied for funding amendment requests or sought to finalize evaluation plans and funding agreements with PHAC. Other studies have found research with marginalized communities often faces similar ethics approval delays due to lack of representation within institutional review boards (IRBs), ideological assumptions surrounding vulnerability and divergent understandings of risk between researchers, participants and IRB boards.^16^,^17^ IRBs have also been critiqued for institutionalizing “colonial unknowing” by separating risk from structural context.^18^ Future funding may seek to interrogate the increased onus placed on research with Black communities. It may also make these processes more streamlined, and lower barriers. 

Pandemic impacts demanded that organizations adapt by moving services online, changing honorariums and incentives and shifting service provision to address new mental health challenges. Our findings largely support prior work around pandemic interruptions and the potential to learn from flexible funding and service delivery during crises.^19^ They also highlight the significance of inequities in the social determinants of health by showing how inequities such as poverty, racial discrimination and homophobia interwove to shape the experiences and needs of organizations’ client populations. However, it is also important to consider how flexibility is necessary outside of global crises. As we learned from organization 012, programming is often being created in spaces where none had previously existed for Black Canadians. There has been a lack of representation in mental health care and a systemic lack of care for this population. In fact, the absence of an informed, antiracist and culturally responsive approach to mental health care for Black Canadians led to the development and implementation of the MHBC. 

Mainstream service provision and medical research began considering the connections between systemic racism and health more strongly following the murder of George Floyd by Minneapolis police.^20^ Some studies have linked experiences with racial discrimination as a key factor contributing to mental health problems.^21^ Others have focussed on ways systemic racism within healthcare (including provider prejudice) prevent many racialized people from accessing services.^22^ Our findings should refocus funders’ attention on the ways that the over-representation of Black people in the criminal justice system and parallel under-representation of Black people in restorative justice programs, a lack of affordable housing, low income, and systemic racism, homophobia and systems of gender are all key determinants of mental health. 

The MHBC emphasized the importance of partnerships and collaboration. Here, the initiative may need to offer organizations greater support. Maintaining partnerships during the pandemic was cited as a key challenge by four organizations and others explained they had difficulty forming or maintaining partnerships due to lack of reputation, divergent vision and staff turnover. This may be a gap that future funders can consider supporting in nonmonetary ways. For example, funders who have established reputations, such as PHAC, may broker these reputations to facilitate meetings between organizations and potential partners. 

Considering these facilitators of success and the challenges faced, there are important lessons to be learned, and ways in which funders can improve funding support moving forward. First, longer term and more flexible funding may help build capacity in permanent ways. Second, by considering the context within which many Black organizations are operating, funders may better understand how historical and current funding scarcity pits organizations against one another and is not conducive to partnership and collaboration. Though Black leadership was an important factor in organization successes, a lack of Black representation among funder staff was a challenge for several organizations. We heard that divergent expectations around data collection and a lack of Black third-party evaluators made some organizations feel as though they were being micromanaged in ways non-Black organizations might not be. Third, participants advised that funders should support work that increases antiracist competencies among mainstream service providers. 

***Strengths and limitations***

Strengths of this study include a two-stage process whereby researchers first conducted a content analysis of annual and final reporting provided by organizations that had received funding through the MHBC. This allowed us to refine our interview guide and gather greater information about key findings from these documents. However, the study was limited by the fact that some organizations did not provide annual reports from all years, and only three organizations had final reports available at the time of the study. Further, representatives from only 9 out of the total 14 organizations agreed to interviews (one organization had two participants from two different projects). Despite these limitations, interview analysis did reach data saturation and we believe we gained a holistic view of the implementation of the MHBC. 

## Conclusion

The MHBC offered multiyear funding to organizations to develop Black-focussed mental health programs in Black communities across Canada. This study used an embedded case study research design to explore the experiences of organizations that received this funding. We found that organizational success was facilitated by providing honorariums and incentives, by organizing programming through PAR design and through Black leadership. Challenges faced by organizations through this period included delays, impacts of the COVID-19 pandemic, structural violence and community-specific challenges, and difficulties maintaining partnerships.

Funders can better support the provision of participant honorariums and incentives, continue to support PAR design and other tools that enable organizations to respond to shifting community needs, continue to support Black leadership and diversity in program management, assist organizations experiencing delays with obtaining ethics approvals, offer greater assistance during crises such as we experienced during the onset of the COVID-19 pandemic, better consider issues related to systemic racism and structural violence, offer longer term stability within organizations through long-term and flexible funding and seek greater Black representation among funding agencies.

In addressing barriers, a critical race theory intersectional analysis can be helpful.^23^-^25^ A critical race theory intersectional analysis shifts from the individual cultural competency frame to addressing structural issues, and refutes two liberalist claims about the law or policies: (1) that it is colour blind and (2) that colour blindness is superior to race consciousness. Keeping central a critical race theory intersectional competency analysis for Black people will steer the conversation into challenging the anti-Black racism, cisnormative and Eurocentric mentality endemic in social services. 

Black LGBTQ and nonbinary people find themselves at the intersection of multiple and compounding forms discriminations, such as anti-Blackness and anti-LGBTQ sentiments. There is, therefore, a need for funders to be intentional about improving research, healthcare delivery, laws and policies that address the intersectional issues for Black LGBTQ persons, as well as those who are nonbinary, seniors, those experiencing homelessness, persons with disabilities, and the incarcerated as we explore the intersections of race and sexual orientation for Black communities.^26^-^29^ Black communities research in all fields warrants and deserves complexity. Refusing to recognize these intersectional complexities and the heterogeneity of Black people only denies us our humanity.

There are deeply engrained, cisgender, compulsory, heteronormative assumptions that there are only two sexes (male and female) and only two corresponding genders (man and woman); that to be cisgender is the only “natural and normal” gender modality; and that deviations from the status quo are rare, exceptional and aberrant.^28^ Along with this, funders need to better acknowledge anti-Black racism and anti-Blackness and generate disaggregated race-based data to allow for a better understanding of the link between anti-Black structural racism and social determinants of health. Collaborating with and co-producing knowledge with Black communities in a project of liberation can further advance mental health outcomes.

## Acknowledgements

The authors acknowledge the funding support from Public Health Agency of Canada to complete this project. The Public Health Agency of Canada had no influence on the data analysis, interpretations or conclusions made in this project.

## Conflicts of interest

Dr. Ifrah Abdillahi was an employee of the Public Health Agency of Canada at the time of this research. She supported conceptualization and participant recruitment for the project but had no access to confidential information.

## Authors’ contributions and statement

BS: conceptualization, data curation, funding acquisition, methodology, project administration, supervision, validation, writing—original draft, writing—review and editing.

MT: formal analysis, methodology, validation, writing—original draft.

IA: conceptualization, data curation, funding acquisition, project administration, resources, writing—review and editing.

WC: conceptualization, funding acquisition, methodology, writing—review and editing.

The content and views expressed in this article are those of the authors and do not necessarily reflect those of the Government of Canada.

## References

[B01] Olanlesi-Aliu A, Alaazi D, Salami B (2023). Black health in Canada: protocol for a scoping review. JMIR Res Protoc.

[B02] Lucente G, Kurzawa J, Danseco E (2022). Moving towards racial equity in the child and youth mental health sector in Ontario, Canada. Adm Policy Ment Health.

[B03] Kemei J, Tulli M, Olanlesi-Aliu A, Tunde-Byass M, Salami B (2023). Impact of the COVID-19 pandemic on Black communities in Canada. Int J Environ Res Public Health.

[B04] Fante-Coleman T, Jackson-Best F (2020). Barriers and facilitators to accessing mental healthcare in Canada for Black youth: a scoping review. Adolesc Res Rev.

[B05] Promoting health equity: Mental Health of Black Canadians initiative – Mental Health of Black Canadians Fund [Internet]. Government of Canada.

[B06] Budiyanto C, Prananto A, Tan FT (2019). Designing embedded case study research approach in educational research. Int J Pedag Teach Educ.

[B07] Krippendorff K (2019). Content analysis: an introduction to its methodology. Sage publications.

[B08] Braun V, Clarke V (2014). What can “thematic analysis” offer health and wellbeing researchers. Braun V, Clarke V.

[B09] Howells A, Aquino EN, Bose D, et al, study UK (2023). Demonstrating the learning and impact of embedding participant involvement in a pandemic research study: the experience of the SARS-CoV-2 Immunity & Reinfection Evaluation (SIREN) study UK, 2020-2023. Demonstrating the learning and impact of embedding participant involvement in a pandemic research study: the experience of the SARS-CoV-2 Immunity & Reinfection Evaluation (SIREN) study UK, 2020-2023. Res Involv Engagem.

[B10] Abdelazeem B, Abbas KS, Amin MA, et al (2022). The effectiveness of incentives for research participation: a systematic review and meta-analysis of randomized controlled trials. PLoS ONE.

[B11] Head E (2009). The ethics and implications of paying participants in qualitative research. Int J Soc Res Methodol.

[B12] Garnett A, Northwood M (2022). Recruitment of community-based samples: experiences and recommendations for optimizing success. Can J Nurs Res.

[B13] Cornish F, Breton N, Moreno-Tabarez U, et al (2023). Participatory action research. Nat Rev Methods Primers.

[B14] Brathwaite A, Versailles D, di-Hope DA, et al (2022). Black nurses in action: a social movement to end racism and discrimination. Nurs Inq.

[B15] Leitch S, Corbin JH, Boston-Fisher N, et al (2021). Black Lives Matter in health promotion: moving from unspoken to outspoken. Health Promot Int.

[B16] McCracken J (2020). Ethics as obligation: reconciling diverging research practices with marginalized communities. Int J Qual Methods.

[B17] Strauss DH, White SA, Bierer BE (2021). Justice, diversity, and research ethics review. Science.

[B18] Sabati S (2019). Upholding “colonial unknowing” through the IRB: reframing institutional research ethics. Qual Inq.

[B19] Gallo HB, Wilber KH (2021). Transforming aging services: area agencies on aging and the COVID-19 response. Gerontologist.

[B20] Dryden O, Nnorom O (2021). Time to dismantle systemic anti-Black racism in medicine in Canada. CMAJ.

[B21] Salami B, Idi Y, Anyieth Y, et al (2022). Factors that contribute to the mental health of Black youth. CMAJ.

[B22] Fante-Coleman T, Jackson-Best F, Booker M, Worku F (2023). Organizational and practitioner challenges to Black youth accessing mental health care in Canada: problems and solutions. Can Psychol.

[B23] Crenshaw K, Gotanda N, Peller G, Thomas K (1995). Critical race theory: the key writings that formed the movement. The New Press.

[B24] Crichlow W Critical race theory: a strategy for framing discussions around social justice and democratic education. Crichlow W.

[B25] Crichlow W, Gaszo A, Kobayashi K (De)colonization, racialization, racism, and Canadian families: relearning through storytelling about lived experience. Nelson.

[B26] Crichlow W (2003). Buller men and batty bwoys: hidden men in Toronto and Halifax Black communities. University of Toronto Press.

[B27] Crichlow W, Ibrahim A, Kitossa T, Smith MS, Wright HK Black gay scholar and the provocation of promotion. University of Toronto Press.

[B28] Mullings DV, Crichlow W, Salami BO, Mullings DV, Clarke J, Adelakun O Black queer identities and pandemic survival experiences in Canada. Demeter Press.

[B29] Crichlow W, Coburn E, Joseph E (2020). The “she-cession” and Black womanhood during the pandemic: what is to be done. Crichlow W, Coburn E, Joseph E.

